# An examination of mental health policy implementation efforts and the intermediaries that support them in New Zealand, Canada and Sweden: a comparative case study

**DOI:** 10.3389/frhs.2024.1371207

**Published:** 2024-08-21

**Authors:** Heather L. Bullock, John N. Lavis, Gillian Mulvale, Michael G. Wilson

**Affiliations:** ^1^ Department of Health Research Methods, Evidence and Impact (HEI), McMaster University, Hamilton, ON, Canada; ^2^ Waypoint Research Institute, Waypoint Centre for Mental Health Care, Penetanguishene, ON, Canada; ^3^ DeGroote School of Business, McMaster University, Hamilton, ON, Canada

**Keywords:** evidence-informed policy, implementation science, mental health, addiction, intermediary, case study, technical assistance, policy implementation

## Abstract

**Introduction:**

The implementation of evidence-informed policies and practices across systems is a complex, multifaceted endeavor, often requiring the mobilization of multiple organizations from a range of contexts. In order to facilitate this process, policy makers, innovation developers and service deliverers are increasingly calling upon intermediaries to support implementation, yet relatively little is known about precisely how they contribute to implementation. This study examines the role of intermediaries supporting the implementation of evidence-informed policies and practices in the mental health and addictions systems of New Zealand, Ontario, Canada and Sweden.

**Methods:**

Using a comparative case study methodology and taking an integrated knowledge translation approach, we drew from established explanatory frameworks and implementation theory to address three questions: (1) Why were the intermediaries established? (2) How are intermediaries structured and what strategies do they use in systems to support the implementation of policy directions? and (3) What explains the lack of use of particular strategies? Data collection included three site visits, 49 key informant interviews and document analysis.

**Results:**

In each jurisdiction, a unique set of problems (e.g., negative events involving people with mental illness), policies (e.g., feedback on effectiveness of existing policies) and political events (e.g., changes in government) were coupled by a policy entrepreneur to bring intermediaries onto the decision agenda. While intermediaries varied greatly in their structure and characteristics, both the strategies they used and the strategies they didn't use were surprisingly similar. Specifically it was notable that none of the intermediaries used strategies that directly targeted the public, nor used audit and feedback. This emerged as the principle policy puzzle. Our analysis identified five reasons for these strategies not being employed: (1) their need to build/maintain healthy relationships with policy actors; (2) their need to build/maintain healthy relationships with service delivery system actors; (3) role differentiation with other system actors; (4) perceived lack of “fit” with the role of policy intermediaries; and (5) resource limitations that preclude intensive distributed (program-level) work.

**Conclusion:**

Policy makers and implementers must consider capacity to support implementation, and our study identifies how intermediaries can be developed and harnessed to support the implementation process.

## Introduction

The implementation of evidence-informed policies and practices (EIPPs) at scale across whole systems is a complex, multifaceted endeavor. Yet an effective implementation process is critical in bridging the gap between the promise of EIPPs and positive outcomes for citizens and society. This is particularly true when the EIPP is psycho-social in nature requiring the mobilization of multiple organizations, often multiple roles within organizations, a need to respond to the diversity of individuals or families receiving the EIPP, and a need to take into account a range of contexts. It is this complexity that may account for the continued lack of access to psycho-social EIPPs for both adults and children. For example, in the US, researchers found that the overall penetration rates for six behavioural evidence-based treatments was only 1%–3% and adoption rates were static or declining across the states who had invested in them (1). This is despite an increased understanding of the burden of mental illness and addictions (2) and increased momentum by policy makers around the globe to address the issue (3).

In response to these challenges, policy makers, innovation developers and service deliverers are increasingly looking toward organizations or programs that can facilitate the implementation process. These organizations are often referred to as intermediaries. Intermediaries act as “translators” for EIPPs and provide technical assistance to organizations and providers that deliver services for citizens, while informing policy and systems (4–7). In general, intermediaries fall under the broader implementation construct of facilitation (8, 9) or change agency (10) with the recognition that complex change processes, such as implementing a new EIPP, do not on their own reach a high enough rate of penetration and fidelity in systems to produce their intended benefits. In order for this to happen, external supports are typically required and intermediaries are one way through which facilitation can take place.

Limited research exists on this type of intermediary and there is not yet a consensus on what precisely defines them and how they contribute to implementation. One reason for this is that the scholarship that exists comes from different fields (e.g., public management, social sciences or implementation science), which naturally draw from different theories, methods and ways of reporting. Added to this is a great deal of heterogeneity in terms of topics such as: child, youth and family services (5, 11), education (12, 13), environment (14), mental health and addictions (15, 16), occupational health and safety (17) and technology (18), where the contexts surrounding the intermediaries vary, limiting the comparability across them. Finally, there are a diversity of terms in use, with some of the more common including: intermediary (organization), purveyor, technical assistance center, knowledge brokering organization, centre of excellence, implementation team and backbone organization (19–26). This lack of precision means that different terms may be used to describe similar constructs and the same term may also be used to describe two quite different constructs, leading to further conceptual fuzziness.

The strategies employed by intermediaries vary but the existing literature does point to some common strategies and approaches. A survey of 68 intermediaries found support for seven core functions of intermediaries, including: consultation activities; best practice model development; purveyor of evidence-based practices; quality assurance and continuous quality improvement; outcome evaluation; training, public awareness and education; and policy and systems development (27). More recently, a web scan and survey of child behavioral health intermediaries found that they used an average of 32 distinct strategies to implement evidence-based interventions, with common strategies including educational, planning and quality improvement strategies (15). They found little consensus, however, on which strategies intermediaries perceived as the most effective.

Some authors frame the strategies of intermediaries in different terms. For example, they describe the approaches of intermediaries and other “support system infrastructure” as including both general capacity-building approaches as well as those that are innovation-specific (28), while others identify strategies targeting different levels in the system (e.g., federal, province/state, local) (7). Still others have described intermediaries in economic terms, suggesting intermediaries can address research supply-side issues (supporting the production, translation and consumption of research) as well as the demand-side issues (such as improving service delivery readiness for a particular EIPP, support for implementation, etc.) (13). To our knowledge, the literature has not distinguished intermediaries based on their public vs. private sector placement.

We identified three sub-types of intermediaries in the literature that specifically address the knowledge production-to-implementation continuum: (1) those whose focus is mainly on translation and dissemination of research evidence to inform policy and practice (knowledge translation-focused, or “KT intermediaries”) (11, 12, 14, 29, 30); (2) those whose focus is mainly on the implementation of pre-packaged research evidence to service providers in the form of evidence-based practices (practice-focused, or “practice intermediaries”) (15, 16, 31); and (3) those whose focus is mainly on assisting policy makers or other system leaders in getting EIPPs embedded at scale in systems (policy-focused, or “policy intermediaries”) (13, 32–34). Of course, many intermediaries will engage in activities across all three types, but this characterization may help to clarify the starting point, goals and theories of change related to each.

Given the focus here on policy and supporting implementation at scale in mental health and addictions systems, our study targets the policy intermediary sub-type. We adopted a definition that we first forwarded by Bullock & Lavis (2019): Intermediaries are organizations or programs that have an explicit and recognized role to support the implementation of government mental health and addictions policy goals and employ specific methods of implementation support. In order to achieve these goals, other actors in the system must understand and accept this role, including those in government, service delivering organizations and other stakeholders.

This study examines the role of policy intermediaries supporting the implementation of evidence-informed policies and practices in the mental health and addictions systems of high-income countries. Guided by implementation theory and drawing from established explanatory frameworks, we address three questions: (1) Why were the intermediaries established? (2) How are intermediaries structured and what strategies do they use in systems to support the implementation of policy directions? and (3) What explains their lack of use of particular strategies?

## Methods

### Integrated KT approach

This study was designed and conducted in collaboration with the International Initiative for Mental Health Leadership (IIMHL)—an international collaborative that focuses on improving mental health and addictions services in eight countries: Australia, Canada, England, Ireland, New Zealand, Scotland, Sweden, and USA (a ninth country, the Netherlands, joined after data collection began). Prior to initiating the study, one of the authors (HB) had been participating with a sub-group of individuals from IIMHL countries who were either working in intermediaries or interested in harnessing the capacity of intermediaries to support systems change. With those relationships in mind, we asked the IIMHL if they would like to partner on this research in an integrated knowledge translation capacity. Integrated knowledge translation (IKT) is an approach to research where those who produce research and those who may use it, partner on a study with the goal on enhancing relevance and facilitating use (35). In this case, our IIMHL partners have thus far participated in three study phases: (1) providing input into the conceptualization and planning of the study, (2) assisting with recruitment and data collection by offering to host the research team during site visits and identifying potential key informants to be interviewed, and (3) assisting with the interpretation of findings and identifying next steps.

### Study design

We used the holistic multiple case study approach outlined by Yin (36). A multiple case study approach is often considered more compelling and robust than single case designs because of the replicative nature and the ability to make predictions from theory that can be tested across cases leading to higher explanatory power. It is a suitable methodology for our questions as it allows for an examination of intermediaries in their context. We brought a realist-postpositivist philosophical approach to this research, considering it a form of empirical inquiry and focusing on maintaining objectivity through the use of techniques like triangulation to minimize errors and get as close as possible to the “truth” (37).

Ethics approval for this study was granted by McMaster University through the Hamilton Integrated Research Ethics Board and informed consent was sought and provided by all participants. The study was conducted in two phases: (1) case selection, and (2) comparative case study. For brevity, we refer to mental health and addictions as “mental health”.

### Phase 1—case selection

Qualitative description was selected as the analytic approach for this phase, which has, as its goal, a comprehensive summary of events in everyday terms (38). The “case” or unit of analysis in this study is defined as: a political jurisdiction with a governing authority that has the ability to develop, implement and evaluate mental health policy and the organizations or programs within them that support policy implementation. This definition means the units may be at different policy levels in systems (e.g., national, provincial/state or municipal). The “population” of potential jurisdictions included countries that are members of the IIMHL. These countries all have well-established health systems and their participation in the IIMHL reflects a commitment to mental health systems improvement and advancement. They provide adequate variation in terms of health service structures, including how mental health services are designed, managed and delivered. They also vary in the factors that may impact successful implementation but have enough similarity to ensure the case study is sensitive to the variables of interest.

The research team worked with IIMHL partners to generate a purposive sample of potential interviewees from each jurisdiction. The list included a mix of leaders in government, agencies of government, non-governmental organizations and service providers who played a leadership role in implementation and could speak to the macro-context of their mental health system. From this list, the research team (HB) contacted one or two leaders from each jurisdiction requesting a brief semi-structured phone interview by telephone or Skype. The questions were targeted toward understanding the policy priorities currently being implemented and the structures in place supporting their implementation. A number of potential interviewees were known to HB through their mutual involvement in the IIMHL.

Interviews were recorded and reviewed by the study team. Using qualitative content analysis and following the qualitative description approach, analysis remained “close” to the data with minimal interpretation. Structured summary sheets of each interview outlining important characteristics and infrastructure were generated and a table was created to facilitate case selection.

### Phase 2—comparative case study

Cases for the comparative case study were purposively sampled based on findings from Phase 1 using an approach that approximates the Most Different Systems design or Mill's Method of Similarity (39). Using this method, cases are selected based on a similar outcome or dependent variable but are diverse in other ways. In this study, cases were selected based on the presence of at least one organization or program that has an explicit role supporting mental health policy implementation (policy intermediary). Cases were also sampled for diversity in other domains such as the policy level (state/province vs. national); mental health system factors (e.g., a range of governance, financial and service delivery arrangements); and, political system characteristics (e.g., diversity in the institutional arrangements, interests and ideas at play) (Table 1). Using this approach, the cases selected include: New Zealand, the province of Ontario in Canada, and Sweden. Ontario included three embedded cases. The cases are bounded in two ways. First, by the political areas specified above that have policy authority over mental health and addictions. Second, they are bounded temporally, that is, this research only considers active implementation efforts and the current structures in place to support them and does not look explicitly at past policy efforts.

**Table 1:** Case selection criteria by jurisdiction.

Jurisdiction	Sub-jurisdiction	Jurisdiction type and population (2015)	Welfare state regime type^b^	Identified structure(s) for policy implementation	Used explicit implementation methods	Receptivity of local stakeholders^c^ (1–3)	Similarity of system structure to Ontario	Notes
Australia		Country 23.13 million	Liberal	✓^a^	✗	2	High	Most activity not at national level
	New South Wales	Province/State 7.54 million	Liberal	✓^a^	✗	2	High	Did not participate in interviews
Canada	Ontario	Province/State 13.6 million	Conservative	✓	✓	3	High	Have connections to stakeholders
	Saskatchewan	Province/State 1.13 million	Conservative	Mandate not renewed	✓	3	High	Mandate not renewed for I-Team
England		Country 53.01 million	Liberal Subgroup	✗	✗	2	Med	No current structures with this focus
Ireland		Country 4.60 million	Liberal Subgroup		✗	2	Med	Informal structures contracted for some work
New Zealand		Country 4.47 million	Liberal Subgroup	✓	✓	3	Med	Clearly defined structure
Scotland		Country 5.30 million	Liberal Subgroup	✓	✓	1	Med	No contact with system leaders
Sweden		Country 9.59 million	Social Democratic	✓	✓	3	Med/Low	Clearly defined entity but re-structuring
USA		Country 318.9 million	Liberal	✓	✓	2	Low	Mix of structures across system
	New York City	City 8.55 million	Liberal	✓	✓	3	Low	New structures in place

^a^ Structures to support system oversight in form of mental health commissions, but not identified in interview.

^b^ Bambra (2005) compares countries based on health care services and decommodification.

^c^ Receptivity Scale: 1 = no contact or low receptivity; 2 = some contact and some either some receptivity OR have not asked directly OR consent form indicates interest in being approached**;** 3 = frequent contact or have asked directly and received positive response.

The methods used for this phase included an analysis of key documents, site visits and follow-up interviews. Field notes were also recorded throughout the site visit by the study team.

#### Review of key documents

We analyzed key documents collected as part of case selection and additional documents retrieved through web searches of government and stakeholder websites and a search of PubMed, Google Scholar and LexisNexis in October 2016 and again in June 2018 for relevant research articles and media accounts related to the intermediaries or implementation efforts. The types of documents analyzed include: annual reports, government reports, news articles, KT products produced by intermediaries and peer reviewed research. Documents were reviewed and data were extracted based on the following domains: health system and political system characteristics; intermediaries and other structures supporting implementation of mental health and addictions priorities; and implementation strategies being utilized.

We reviewed and analyzed a total of 73 sources: 24 policy documents, 13 reports or other documents generated by or on behalf of the intermediary, 22 websites and 14 scholarly publications. We also reviewed some grey literature on implementation infrastructure that referenced at least one of the cases (*n* = 3) and used news media articles as a source of triangulation to verify events that were mentioned by stakeholders during the interview (Appendix 1). We used each intermediary's website to review reports and publications, so many of those are not counted in the tally above.

#### Site visits

Our team created a matrix outlining the types of stakeholders we wanted to interview and shared it with the IIMHL IKT partners in each jurisdiction. Partners were instructed to identify at least two individuals for each category and provide contact details. Types of stakeholders included: (1) intermediary, (2) policy makers/government, (3) funder(s) of implementation/intermediary, (4) oversight of implementation/intermediary, (5) researchers familiar with the intermediary, (6) knowledge synthesizers & translators, (7) recipients of implementation supports, (8) partners of intermediary, and (9) others. One to two people from each category were then invited to participate. The consent form was translated into Swedish for the Swedish case, and while the interviews were conducted in English, an informal English/Swedish interpreter (someone who was familiar with the subject) was offered to potential participants.

Interview questions were tailored to the type of stakeholder but were focused on constructing a full picture of how policy implementation is structured and delivered in the system, including: (1) what policy priorities are currently being implemented; (2) who (organizations and individuals) are supporting their implementation; (3) what implementation strategies they use (e.g., training, audit and feedback, etc); (4) how the implementation supports are valued and meeting the identified goals; and (5) what factors were important in the creation of the intermediary (Appendix 1). The interview guide was revised as the analysis of earlier rounds of data proceeded and theoretically or substantively important insights were identified for exploration in later rounds. With consent, interviews were recorded for later transcription and lasted approximately 90 min each. Interviews were conducted until saturation was reached and no key perspectives were deemed missing. Throughout the site visit, the study team took field notes including descriptive (e.g., who, what, where, etc.) and interpretive information (e.g., personal reflections and questions arising from activities). Additional documents, such as presentations or reports, were requested from participants and reviewed. All site visits took place in 2017: New Zealand (February), Sweden (May) and Ontario (July–September). When appropriate based on the rules of the jurisdiction, ethics waivers were sought and acquired prior to the site visit.

#### Follow-up interviews

A final stage of data collection included interviews with key informants who were unable to participate during the site visits or agreed to a follow-up interview as analysis proceeded. These additional interviews took place in 2017 and 2018. This was done to ensure each case was as complete and as comparable as possible across jurisdictions.

A total of 49 initial interviews were conducted during the site visits or shortly thereafter (13 NZ, 23 ON, 13 SE). More interviews were conducted in Ontario because the three embedded cases meant that a larger sample of stakeholders were required to reach saturation. Three of the interviews in Sweden were supported by an interpreter. Stakeholders from all of the categories identified in the stakeholder matrix were interviewed for each case, providing us with a well-rounded perspective. Four follow-up interviews were also conducted to confirm details or fill small gaps in the analysis.

NVivo12 Qualitative Software was used to manage data, thereby serving to establish a comprehensive and easily accessible case study database.

### Analysis

Transcripts and/or audio recordings were reviewed at least twice. Supporting documents were also reviewed and coded. Directed content analysis (40) was employed, which begins the coding process by drawing from existing research and theory as a guide. Within each case, sources were compared with one another to identify themes that emerge across them. The lead researcher (HB) led all stages of the analysis and JNL, GM and MW were involved in reviewing codes, themes and interpretation.

### Analytic goals and frameworks

#### Goal 1

To explain why the intermediaries were originally established and endorsed by governments to support policy implementation, we used Kingdon's multiple streams agenda-setting framework (41). Kingdon's theory identifies activities in independent “streams” that have to come together during a brief “window of opportunity”. These include: heightened attention to a problem (problem stream), an available and feasible solution (policy stream), and the motive to select it (politics stream). The three streams must come together in order for a change to be made, and this usually happens through the work of a policy entrepreneur.

Using this framework, we identified the timelines of the relevant events and activities leading up to the establishment of the intermediary(ies) based on stakeholder accounts of what was relevant as well as our document review. Next, we developed a comparative table that highlighted: (1) aspects of the problems in each system that each intermediary was created to address, (2) policy proposals and ideas that were supportive of the need for implementation infrastructure in the form of an intermediary, (3) the political environment that made the intermediary(ies) as a policy solution feasible, and (4) the relevant actors, including policy entrepreneurs that were important for bringing the intermediary to the decision agenda.

#### Goal 2

To describe and compare the structures of the intermediaries, their organizational characteristics and the implementation strategies they use, we drew on a modified version of the Interactive Systems Framework for Dissemination and Implementation (ISF) as a descriptive framework. The ISF was originally developed by Wanderman and colleagues (28, 42) and is a heuristic that captures how new knowledge moves from research development to widespread use and the systems and processes supporting this movement. The ISF specifies the three systems needed to carry out dissemination and implementation functions: (i) Synthesis and Translation System; (ii) Delivery System; and (iii) Support System. In an effort to capture the important role of policy in implementation, we modified the ISF by adding a Policy System (links with the three other Systems and provides a variety of policy-related supports for dissemination and implementation) (Bullock. 2019).

We used the modified ISF to sort and classify the strategies used by intermediaries according to the “target” System. We then added some categories that we felt were important to highlight and did not necessarily fit well within one particular System: strategies targeting the public; strategies targeting individuals with lived experience & family members; and strategies focused on performance assessment and/or system-monitoring. Finally, we cross-referenced our strategies with the implementation strategies identified by Powell and colleagues (43) who used the sub-categories of “Plan”, “Educate”, “Finance”, “Re-structure” “Quality Management” and “Attend to Policy Context”. Next, we extracted examples of the strategies for each case from the interview data, and cross-referenced/supplemented these with the document and website data sources.

#### Goal 3

To explain the choice of implementation strategies we first drew on the 3I + E framework (44, 45). The 3I + E framework is used to explain how Institutions (e.g., government decision-making structures and processes), Interests (i.e., groups with a vested interest), Ideas (i.e., values and research-based knowledge) and External factors (i.e., events outside of the policy area of interest) affect the actions of those making decisions or implementing them.

Our original intent was to use this framework for a complete analysis, however, once we had results from the second question, we found we had a far more interesting policy puzzle related to the lack of use of particular strategies that warranted a slightly different analytic approach including a thematic analysis of salient features that fell under two elements of the 3I + E framework.

### Context: intermediary case descriptions

**Figure 1:**
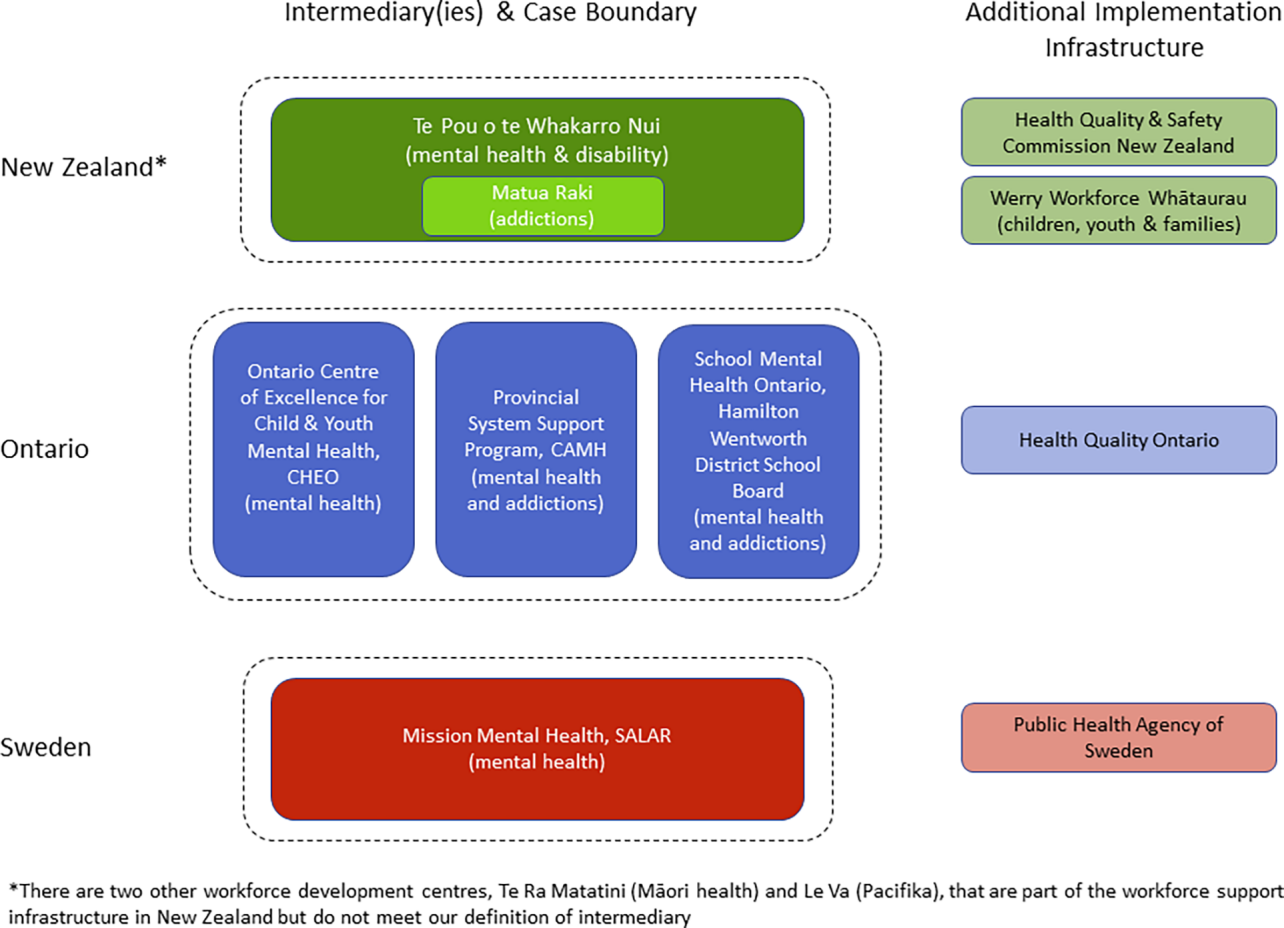
Graphic depiction of implementation support infrastructure by case.

Figure 1 depicts the intermediary infrastructure in each case as well as the case boundaries.

#### New Zealand

The Ministry of Health, through Workforce New Zealand, funds a national infrastructure to support development of the mental health and addictions workforce, including 5 centres with different foci. Over time, Te Pou o te Whakaaru Nui (Te Pou, adult mental health and disability focus) and Matua Raki (addictions focus, housed at Te Pou), have developed into an intermediary that aligns with our definition and is the focus of the NZ case. Two other organizations that are increasingly contributing to the implementation infrastructure include the Werry Workforce Whāraurau (child and youth focus) and the Health Quality & Safety Commission New Zealand.

#### Ontario, Canada

In Ontario, we identified three intermediaries that fit our definition: (1) Ontario Centre of Excellence for Child and Youth Mental Health (OCoECYMH) located at the Children's Hospital of Eastern Ontario and funded by the Ministry of Children and Youth Services (note: post-data collection, funding authority was transferred to the Ministry of Health and Long-Term Care, MOHLTC); (2) Provincial System Support Program (PSSP) located at the Centre for Addiction and Mental Health and funded by MOHLTC; and (3) School Mental Health ASSIST (SMH ASSIST) located at the Hamilton-Wentworth District School Board and funded by the Ministry of Education. These three intermediaries collectively comprise the Ontario case, however, other organizations, such as Health Quality Ontario, were also highlighted as increasingly playing an intermediary function in mental health.

#### Sweden

Uppdrag Psykisk Hälsa (Mission Mental Health) is the intermediary in Sweden that met our definition and is the focus of this case. Mission Mental Health is located at the Swedish Association of Local Authorities and Regions (SALAR), which is a peak body that acts as both an employers’ organization as well as one that represents the interest of the municipalities and regions to the national government. Mission Mental Health is funded through an agreement between SALAR and the Ministry of Health and Social Affairs. The Public Health Agency of Sweden was also highlighted as an organization beginning to take on more of an intermediary function.

It should be noted that the lead researcher (HB) previously worked with PSSP and has pre-existing relationships with all three intermediaries in Ontario.

## Results

### Why were the intermediaries established?

Table 2 identifies the timelines of the relevant events and activities leading up to the establishment of the intermediary(ies) based on stakeholder accounts and our document review. The results of the analysis of factors influencing the decision to establish the intermediaries is presented in Table 3.

**Table 2:** Timelines of events leading up to the establishment of the intermediaries for each case.

Events and activities by case				
New Zealand Te Pou (est. 2006)	Ontario, Canada			Sweden Mission Mental Health (est. 2008)
	OCoECYMH (est. 2004)	PSSP (est. 2011)	SMH Assist (est. 2011)	
**1990s**. A number of “dreadful events” involving people with mental illness	**1999.** Mental Health Implementation Task Forces initiated			**1994.** Government Bill 1993/94:218—*Mentally Ill People's Conditions* identifies separation of care for mental health between counties & municipalities
**1993**. Dr Janice Wilson becomes Director of Mental Health in Ministry of Health	**1999.** *Making It Happen: Implementation plan for mental health reform* published by government			**Early 2000s**. Shift in technology and thinking fostered demand for new ways of thinking and doing things
**1995**. Judge Kenneth Mason leads national inquiry and publishes findings	**1999.** 2 provincial standard outcome measures announced for children & youth mental health services			**2003.** Murder of Anna Lindh, Swedish Foreign Minister and several other acts of violence involving people with mental illness
**1996**. Government passes Mental Health Act (1992) replacing Lunatics Act (1882)	**2000.** Ontario Health Services Restructuring Commission recommends reforms to mental health services (*Looking Back, Looking Forward, 2000*)			**2003.** National review of mental health led by Dr Ing-Marie Wieselgren and Anders Milton (2003–2006)
**1997**. Establishment of Mental Health Commission	**2002.** New premier looking to retain office			**2006.** Commission presents its final inquiry report to government, becoming an important knowledge base for future government activities
**1998**. Mental Health Commission publishes Blueprint 1	**2002.** *The Time is Now: Themes and recommendations for mental health reform in Ontario* Final Report of the Provincial Forum of Mental Health Implementation Task Force Chairs			**2006.** New government with focus on performance-based reimbursements Lyons/Alliance government, including appointment of Goran Hägglund as Minister of Health and Social Affairs
**1999—early 2000s**. Government (through Treasury) willing to invest heavily in mental health	**2002.** 1st comprehensive epidemiological reports published on child & youth mental health in Canada *(Waddell, 2002; Health Canada 2002)*			**2007.** Swedish Association of Local Authorities & Regions (Sveriges Kommuner och Landsting) was created as a coordination body between national and regional/municipal levels of government
**2001**. Ministry of Health announces funding for 2 workforce development initiatives	**2003.** ON Auditor General's report identifies major concerns in children & youth mental health			**2007.** National government institutes new way of supporting mental health by contracting directly with local authorities and regions
**2002**. Ministry of Health publishes *Mental Health (Alcohol and Other Drugs) Workforce Development Framework*, acknowledging a more systemic approach to workforce development is required	**2003.** Government announces intention to create a centre of excellence for children's mental health at Children's Hospital of Eastern Ontario			**2008.** Government communication document 2008/09:185—*A policy for people with mental illness or mental disability*
**2002**. Health Research Council begins to run adult mental health workforce programs	**2003.** Election & change in government			**2008. Mission Mental Health (Uppdrag Psykisk Hälsa) at SALAR is established**
***2003**. Werry Centre for Child and Adolescent Mental Health launched by Minister of Health (Annette King) at University of Auckland*	**2004. The Provincial Centre of Excellence for Child and Youth Mental Health at CHEO established**			
**2003–2004**. First national epidemiological survey/report on mental health and addictions *Te Rau Hinengaro—The New Zealand Mental Health Survey*	**2006.** Ministry of Children and Youth Services publishes *A shared responsibility: Ontario ’s policy framework for child and youth mental health*.			
**2005**. Ministry of Health publishes the second mental health and addiction plan: *Te Tāhuhu: Looking forward, moving forward Improving mental health 2005–2010*	**2006.** Canadian senate committee publishes *Out of the Shadows at Last: Transforming mental health, mental illness and addiction services in Canada,* Kirby & Keon			
**2005**. Health Workforce Advisory Committee publishes *Strategic Principles for Workforce Development in New Zealand*	**2007.** Mental Health Commission of Canada is established			
**2005**. *Tauawhitia te Wero Embracing the Challenge* National mental health and addiction workforce development plan 2006–2009 is published by Ministry of Health	**2009.** Minister's Advisory Group publishes *Every Door is the Right Door* discussion paper and 5 theme group papers			
**2006**. Ministry of Health publishes implementation plan for Te Tāhuhu: *Te Kōkiri—The mental health and addiction action plan 2006–2015*	**2009.** OCoECYMH contracts a policy-oriented paper on school-based mental health: **2009.** *Taking Mental Health to School: A policy oriented paper on school-based mental health for Ontario* (authors include Kathy Short)			
**2006. Te Pou o te Whakarro Nui is established**	**2010.** Minister's Advisory Group publishes *Respect, Recovery, Resilience: Recommendations for Ontario's Mental Health and Addictions Strategy* final report			
	**2010.** All-party committee submits final report *Navigating the Journey to Wellness: The Comprehensive Mental Health and Addictions Action Plan for Ontarians*			
	**2010.** Ministry of Education requests proposal from Kathy Short (for what later becomes SMH ASSIST)			
	**2011.** Government publishes *Open Minds, Healthy Minds, Ontario's 10-year mental health and addictions strategy*			
	**2011. Provincial System Support Program at CAMH is established**			
	**2011. School Mental Health ASSIST at Hamilton-Wentworth District School Board is established**			

Bolded text highlights when each intermediary was established.

**Table 3:** Factors that influenced the decision to create intermediaries, drawing from the Multiple Streams framework (41).

Factors	Description of factors that influenced decision to create intermediaries by case				
	New Zealand Te Pou (est. 2006)	Ontario, Canada			Sweden Mission Mental Health (est. 2008)
		OCoECYMH (est. 2004)	PSSP (est. 2011)	SMH Assist (est. 2011)	
**Problems stream**	**Focusing Events** A number of “dreadful” events involving people with mental illness happened with a lot of public attention in 1990s **Feedback About a Problem/Change in Indicator** First national epidemiological study conducted, that shed light on the full scale of the problem (mental health issues)	**Feedback About a Problem/Change in Indicator** Visibility about mental health increasing in general (internationally, nationally and provincially) and children and youth mental health in particular Federal Senator Michael Kirby labels children's mental health as the most neglected area of health care and dubs it “*the orphan of the orphan*” Government elites needed to be perceived as investing on the heels of hospital amalgamations, including changes to mental health services *ON Auditor General 2003* identified many problems in child & youth mental health Key study (Waddell et al. 2002) and key report (Health Canada 2002) identified scale and scope of child and youth mental health problems in Canada	**Feedback About a Problem/Change in Indicator** ***Government*** Receiving feedback through Select Committee on Mental Health and Addictions and other government activities that people were ‘falling through the cracks’ of systems when transitioning between them (e.g., from child and youth to adult services etc) ***CAMH*** New CEO looking to restructure the organization and was getting feedback to consider the provincial capacity that was available through policy legacies through the merger of 4 mental health and addictions facilities in Toronto and ensuring it was put to good use	**Feedback About a Problem/Change in Indicator** Provincial government was receiving feedback from multiple directions that more needed to be done to support mental health of children and youth in schools e.g., Mental Health Commission of Canada issued RFP for work on school-based MH in 2008; efforts by OCoECYMH to increase visibility of issue	**Focusing Events** Murder of Anna Lindh, former Swedish Foreign Minister by individual thought to be mentally ill (2003) and several other incidents of harm by persons with mental illness profiled in media around the same time **Feedback About a Problem/Change in Indicator** Government Bill 1993/94:218—*Mentally Ill People's Conditions* identified separation of care for mental health between counties & municipalities. This resulted in problems of coordination across organizations that left gaps in the system. Mental health viewed broadly (not just mental illness)—this view increased visibility of coordination problems across levels of government and sectors
**Policy stream**	A great deal of policy activity in decade before establishment, identifying the need for major system reforms, including an increasing focus on workforce development. Examples: -Mason Inquiry (1996) -*Blueprint 1* (Mental Health Commission, 1998) *-Mental Health (Alcohol and Other Drugs) Workforce Development Framework*, Ministry of Health, 1992) *-Te Tāhuhu: Looking forward, moving forward Improving mental health 2005–*2010 (Ministry of Health, 2005) *Strategic Principles for Workforce Development in New Zealand* (Health Workforce Advisory Committee, 2005)	Activity at the national level (e.g., consultations to develop *Out of the Shadows at Last: Transforming Mental Health, Mental Illness and Addiction Services in Canada* (2006) Final Report of The Standing Senate Committee on Social Affairs, Science and Technology led by Senators Michael Kirby & Wilbert Joseph Leon) and provincially (e.g., Ontario Auditor General's report (1993) increased visibility of the need for changes to the child and youth mental health sector. “Centres of Excellence” as a policy concept was attractive across different policy areas	Respect, Recovery, Resilience: *Recommendations for Ontario's Mental Health and Addictions Strategy* (2010) developed by the Minister's Advisory Group identified need to work across services & sectors. Direct proposal from CAMH to government repositioning some of its capacity as policy implementation support (2010/2011) Ministry of Health & Long-Term Care was looking for implementation partners to support their initiatives in the upcoming 10-year mental health strategy, *Open Minds, Healthy Minds* (2011)	A process that brought policy makers together to support the development of the document: *Taking mental health to school: A policy-oriented paper on school-based mental health for Ontario* (Santor, Short, & Ferguson 2009) increased salience & acceptability of idea Policy documents began to identify schools as a key location to support early identification/ intervention and school graduation rates as key outcome K. Short already running technical assistance centre in HWDSB (government saw idea had credibility and could be scaled) MED sought proposal from Short	Government strikes a *National Coordination of Mental Health Services* Commission led by Ing-Marie Wieselgren and Anders Milton Policy documents identified a need for better coordination across actors and levels of government Policy decision by national government made to contract differently with local authorities and regions for mental health services through direct agreements
**Politics stream**	**Swing in national mood** Increasing visibility of the issue and decrease in stigma created widespread support for investments in mental health **Changes in the balance of organized forces** Formation of Mental Health Commission **Events within government** Treasury willing to make investments in mental health “And, in part, because the money was flowing. The money was really flowing at that point, so we could afford to build infrastructure.” Hired Dr Janice Wilson as Director of Mental Health	**Changes in the balance of organized forces** Hospital amalgamations in early 2000s caused an even greater need for strong community services The striking of mental health implementation task forces engaged stakeholders in solution-finding **Events within government** Government was not polling well and looking to hold power prior to next election through investments after years of cutbacks. This was unsuccessful and the government changed in 2003 but the idea of a Centre of Excellence remained relevant. Lack of opposition to investments in children's mental health (clear “win” and concept of “centre of excellence” was politically palatable)	**Events within government** Striking of All-Party Committee Needed to find partner(s) to support implementation of key policy initiative on transitions between services and sectors Fit—CAMH already had capacity and could get up and running quickly	**Events within government** Striking of All-Party Committee & MAG who were taking a broader perspective on mental health including more focus on prevention/promotion and early intervention Congruent with provincial mood Needed to be seen as doing something regarding mental health in schools	**Swing in national mood** Increased visibility of the issue due to publicity related to Anna Lindh and aided by advances in information technology **Changes in the balance of organized forces** Creation of Swedish Association of Local Authorities & Regions SALAR (Sveriges Kommuner och Landsting as a coordination body between local/regional levels and national government provided natural “home” for an intermediary **Events within government** Health and Social Care minister who was willing to invest and believed that while you can't win an election based on mental health as a policy issue, you can lose one
**Participants**	**Policy entrepreneur** Dr Janice Wilson, psychiatrist and first Director of Mental Health for NZ government **Other visible participants** Judge Kenneth Mason (led 2 inquiries) Barbara Disley (first Mental Health Commissioner)	**Policy entrepreneurs** Dr Simon Davidson, prominent child psychiatrist who was an expert advisor to government on hospital amalgamations related to children's services and considered an innovator in the field Peggy Taillon, key figure in mental health implementation task force work and an advisor to government on this and other health reforms, suggested a “centre of excellence” to government officials **Hidden participants** Dr Ian Manion, CPsyc who became co-executive director of the OCoECYMH Peter Finkle, Regional Director, MOHLTC	**Visible participants** Dr Bob Bell, Deputy Minister of Health Dr Catherine Zahn, President and CEO of CAMH **Hidden participants** Susan Paetkau, MOHLTC Director - key decision maker in appointing PSSP as lead for service collaboratives initiative Susan Pigott, VP at CAMH—reporting line for PSSP and liaison with MOHLTC Dr Nick Kates, physician & member of MAG, originally developed service collaboratives concept	**Visible participants** Dr Kathy Short, school psychologist, and now lead of SMH ASSIST Dr Bruce Ferguson, psychologist, member of the MAG and expert advisor to government **Hidden participants** Barry Finlay, MED Director—key decision maker John Malloy—Director of Education, Hamilton-Wentworth District School Board	**Policy entrepreneur** Dr Ing-Marie Wieselgren, psychiatrist and co-lead of national inquiry. Then became first chief executive for Mission Mental Health **Other visible participants** Dr Anders Milton—prominent physician and co-lead of national inquiry Goran Hägglund—Minister for Health and Social Affairs who understood the political value of the mental health agenda **Hidden participants** Karin Johansson, state secretary, Ministry of Health and Social affairs

Bolding indicates elements drawn from the Multiple Streams framework (41).

In all three cases, the intermediary infrastructure came on the heels of a monumental shift in how mental health and addictions care was delivered—moving from a system of institutional-based care to one based largely in community. While the timelines and trajectories for deinstitutionalization varied across cases (46–51) the process was completed around the turn of the century—and it is in the decade that followed that these intermediaries were established.

The deinstitutionalization process left policies legacies that differed in each case due to the unique political terrain and health policy features of each jurisdiction. However, this shift in the model of care was largely cited by key informants as a factor that was influential in driving the need for new and different capacities in the system as a result of it becoming more complex and multi-faceted and spanning a new array of community and hospital environments. The type of new capacity required was framed differently across cases and is outlined as part of the analysis below.

#### New Zealand

During the years following deinstitutionalization, mental health became a much more visible policy issue due to several “dreadful events” involving people with mental illness and feedback about the scale and scope of the issue from the first national epidemiological study on mental health issues (problem stream). This increased visibility of the problem led to a flurry of a policy activity, including a government inquiry, at least seven policy documents and a major change in the law (policy stream). Also during this time was the formation of a Mental Health Commission and a government that was willing to invest heavily in mental health (politics stream). Over time, some of the challenges identified in the system were framed as a need to expand the workforce to include other roles that were not required in an institutionally-based care model and to simultaneously equip the existing workforce to function differently than they had been expected to in the past.

The policy entrepreneur (Janice Wilson) was recognized by almost all key informants as playing a pivotal role in getting the workforce infrastructure established. However, workforce centres in and of themselves, did not meet our definition of an intermediary. Since their establishment, TePou, Matua Raki and more recently, the Werry Centre, have evolved into the role of an intermediary. This broader role may have been bolstered by the government's decision in 2012 to eliminate the New Zealand Mental Health Commission and transfer only limited functions to the Office of the Health and Disability Commissioner, leaving additional gaps in the system now filled by these intermediaries.

#### Ontario, Canada

In Ontario, the first intermediary to be established was the Ontario Centre of Excellence for Child & Youth Mental Health (OCoECYMH)—almost seven years before the Provincial System Support Program (PSSP) and School Mental Health Assist (SMH ASSIST). Prior to OCoECYMH's creation, there was an increasing visibility of children and youth mental health as an issue that needed to be addressed at the national and provincial levels. For example, a Federal Senator, Michael Kirby, called children's mental health the “orphan of the orphan of health care”. In addition, feedback about the problem in the form of research identifying the true scope of the problem in Canada was developed (problem stream). On the political front, the sitting provincial government was not doing well in the polls and was seeking to gain some positive political momentum in an election year by announcing some investments after several years of cuts (politics stream). Children and youth mental health was identified by the provincial auditor general as an area in need of transformation and after a recent round of hospital amalgamations, mental health interest groups were seeking investment to bolster the community sector. From a policy perspective, certain government insiders had been advancing the concept of “centres of excellence” to address a wide variety of policy areas and a new ministry, Ministry of Child and Youth Services had just been created in 2003 (policy stream). The government then reached out to Simon Davidson and colleagues, inviting them to develop a proposal for a centre of excellence for children and youth mental health. Our analysis suggests that two people, Dr Davidson, a prominent child psychiatrist who had developed close relationships with government officials by participating in the hospital amalgamation decisions, and Peggy Taillon, who was an Advisor to the Premier at the time and was very involved in Ontario's Mental Health Implementation Task Force, acted as policy entrepreneurs.

Interestingly, OCoECYMH is also the sub-case that fits most clearly with the Kingdon framework. It is possible that once one intermediary is established in a system for a particular policy area, the concept of additional intermediary capacity is easier for policy makers to buy into based on the policy legacy established by the first. This may mean that the decisions to create PSSP and SMH ASSIST were less “visible” and political in nature and became more “technical” and bureaucratic. In the case of both PSSP and SMH ASSIST, their function was first proposed by those outside of government (CAMH for PSSP and Kathy Short and the OCoECYMH for SMH ASSIST) as a policy solution that could support the implementation of key policy decisions. These policy “solutions” were proposed at a time when the government was developing a new 10-year strategy for mental health and addictions. Bureaucrats in MOHLTC and MEd took advantage of these policy ideas as part of their ministerial commitment and actions related to the new strategy. In general, our analysis suggests for these later intermediaries, most of the activity leading to the decision was in the policies stream (the government was developing a new policy and needed resources that could be mobilized quickly and with a good likelihood of success) and that the decision to invest in this implementation infrastructure was facilitated by the policy legacy created by the establishment of the first.

#### Sweden

Prior to the establishment of Mission Mental Health, the mental health system in Sweden was in some turmoil due to a highly visible death of a politician by someone with a mental illness as well as some other negative events that were profiled in the media (problem stream). These events increased the visibility of mental health as a policy issue and the government at the time was receptive to further investments in the sector (politics stream). One of the outcomes of this was a national inquiry led by Anders Milton, a prominent politician and Ing-Marie Wieselgren,a prominent psychiatrist and who became the content lead for the inquiry. The inquiry made many recommendations including a need to focus on children and youth, which was seen as a large gap (policy stream). Dr. Wieselgren also acted as the policy entrepreneur, coupling the streams, and once the inquiry work was completed, she became the leader of Mission Mental Health.

Sweden is a good example of how the influence of the policy entrepreneur can continue beyond the decision to establish the intermediary itself. In this case, Dr. Wieselgren was intimately aware of the policy issues based on her work on the national inquiry as well as through her previous roles. She had also established a wide array of relationships with different actors across Sweden. This likely enabled the establishment of Mission Mental Health by increasing its acceptability and ensuring that its work aligned with the policy issues that surfaced during the inquiry.

### How are intermediaries structured and what strategies do they use to support the implementation of policy directions?

The structure and organizational characteristics of the intermediaries are summarized in Table 4. Generally, there is a great deal of variation in the structures and organizational characteristics of the intermediaries in our cases, with differences across most of the domains. Key differences include: the settings in which intermediaries are located (e.g., NGO, service delivery organization or peak organization), the age-related focus of the intermediary (e.g., children & youth, adult, full age continuum), the mandate and how far it extends beyond mental health (e.g., addictions, problem gambling, disability), the primary target audience of the intermediary (e.g., hospital, community, schools or cross-sectoral) and the service model (e.g., centralized or distributed). Each intermediary also has very different stated areas of investment and often focused on quite different EIPPs. They also varied around how closely they drew upon implementation or knowledge exchange models, theories or frameworks to guide their work.

**Table 4:** Structure and organizational characteristics of intermediaries.

	Intermediary				
	Te Pou o te Whakarro Nui (including Matua Raki)	Ontario Centre of Excellence for Child and Youth Mental Health	Provincial System Support Program	School Mental Health Assist	Mission Mental Health
Country	New Zealand	Ontario, Canada			Sweden
Public vs. private	Private, not-for-profit (highly regulated)	Private, not-for-profit (highly regulated)	Private, not-for-profit (highly regulated)	Public (highly regulated)	Public (highly regulated)
Setting	Non-governmental organization (Wise Group)	Service delivery organization (Children's Hospital of Eastern Ontario, CHEO)	Service delivery organization (Centre for Addiction and Mental Health, CAMH)	Service delivery organization (Hamilton-Wentworth District School Board)	Peak organization (Swedish Association of Local Authorities and Regions, SALAR)
Main funding source	National government: NZ Ministry of Health (Health Workforce NZ)	Provincial government: Ontario Ministry of Children & Youth Services^a^ (MCYS)	Provincial government: Ontario Ministry of Health and Long-Term Care	Provincial government: Ontario Ministry of Education	National government: Swedish Ministry of Health and Social Affairs
Focus	Adults and older adults	Children & youth	Youth, adults & older adults	School-aged children & youth	Full age continuum
Boundaries of mandate	Mental health, addictions and disability	Mental health	Mental health and addictions (including problem gambling)	Mental health and addictions	Mental health
Primary target audience	Mental health and addictions workforce (focus on District Health Boards)	Child & youth serving community mental health agencies funded by MCYS	Organizations serving people with mental health and/or addictions problems across sectors	School boards	Cross-sectoral regional and local authorities working with mental health in social care, education and health care
Governance structure	Board of Directors	CHEO's Board of Trustees	CAMH's Board of Trustees	Hamilton Wentworth District School Board of Trustees. Reports directly to Director of Education	SALAR's Board (who report to a congress of politically elected officials) & different political committees
Advisory structure(s)	Clinical Sector Reference Group (27 members, including people with lived experience, family/whanau, service sector leaders, and researchers)	Strategic Advisory Council (12 members, including youth, parents/family members and organizational leaders)	Project-specific advisory structures (e.g., EENet persons with lived experience & family panel, provincial collaborative advisory group)	No formal ongoing advisory structure. With co-creation model, regularly receive input from a range of stakeholders	SALAR steering group comprised of internal and external stakeholders
Size (approx.)	43 people	50 people	150 people	13 people provincially supporting 72 mental health leaders in schools	40 people
Annual budget^b^ (approx.)	$20 million NZD	$5.9 million CAD	$19 million CAD	$2.2 million CAD (does not include funding for mental health leaders)	60 million SEK/5.7 million EUR
# Offices & locations	2 offices (Auckland & Wellington)	1 office (Ottawa)	10 offices (Barrie, Hamilton, Kenora, Kingston, London, Ottawa, Sudbury, Thunder Bay, Toronto Central & Toronto Regional)	1 office (Hamilton)	1 office (Stockholm)
Service model	Distributed (travel as needed, particularly to South Island)	Centralized (travel as needed to other locations)	Highly distributed (less travel required based on number of regional offices)	Highly distributed (coaches located across province; mental health leaders in each school board in province)	Centralized (travel as needed to other locations)
High-level description	National centre of evidence-based workforce development for the mental health, addiction and disability sectors in New Zealand	Drive high-quality child and youth mental health services by setting the bar for excellence and collaborating with others to pursue continuous quality improvement	Works with communities, service providers and other partners across Ontario to move evidence to action to create sustainable, system-level change	Provincial implementation support team designed to help Ontario school boards to promote student mental health and well-being using evidence-based approaches	
Stated goal(s)	To improve the workforce performance of mental health, addiction and disability services	Working to strengthen Ontario's mental health programs and services for all children, youth, families and caregivers	Transforming mental health and addictions systems to improve the lives of Ontarians	Enhance quality and coherence in mental health promotion and prevention programming in schools	Create conditions for a sustainable mental health system by encouraging the improvement and enhancement of services and supports, and by increasing accessibility and equality
Investment areas	1. Practice & leadership 2. Information & outcomes 3. Training & development 4. Workforce planning	1. Support evidence-based practice & knowledge in use 2. Maximize capacity in training, research & evaluation 3. Collaborate with stakeholders	1. Knowledge exchange 2. Implementation 3. Information management 4. Health equity & engagement 5. Evaluation	1. Leadership & guidance 2. Implementation coaching 3. Tailored resources 4. Community of practice	1. Coordinate local improvement work 2. Analysis & implementation of local and regional conditions 3. Support development of data collection template for reporting of data and action plans
Recent EIPP foci	• Reducing the use of seclusion & restraints • Increasing the use of talking therapies • Service user, consumer and peer workforce capacity building • Addressing co-existing mental health and addiction problems • Improving the physical health of people experiencing mental health or addiction problems	• Enhancing family engagement in services • Enhancing youth engagement in services • Improving service quality and performance • Promoting community-based suicide prevention and life promotion through coaching • Coordinating a Lead Agency Community of Practice	• Developing service collaboratives to supports transitions of people across services and sectors • Implementing Ontario Perception of Care Mental Health and Addictions tool • Implementing Staged Screening and Assessment protocol • Supporting knowledge exchange for Early Psychosis Intervention Ontario Network • Developing an Opioid Resource Hub	• Enhancing the organizational conditions for mental health in schools • Improving mental health literacy for educators • Addressing tragic events in schools • Decision support for school boards for mental health programming selection • Life promotion and suicide prevention	• Mental health for asylum seekers and new arrivals • Supporting the implementation of social investment • Workplace mental health • Creation of a multi-region infrastructure for knowledge sharing and improvement • Mental health in schools
Use of knowledge exchange and/or implementation theory to underpin work	No Does not draw for any theory in particular but will integrate concepts as deemed appropriate (e.g., PDSA cycles)	Somewhat • Concept of co-production used in youth and family engagement work • Created toolkits for sector on knowledge mobilization and implementation based on theory	Yes • Network theory (EENet) • NIRN's Active Implementation Frameworks	Yes • Co-production • NIRN's Active Implementation Frameworks	No Does not draw for any theory in particular but will integrate concepts as deemed appropriate (e.g., incorporating IHI's model for improvement)

^a^ In 2018 the Ontario government dissolved the Ministry of Children & Youth Services. Responsibility for this portfolio now rests with the Ministry of Health and Long-Term Care.

^b^ In many cases, the intermediary acts as a flow through for funds to others in the system. The full annual budget is not necessarily retained and used directly by the intermediary.

In terms of similarities, three of the five intermediaries were around the same size (40–50 people), although PSSP was much larger (150 people) and SMH ASSIST was much smaller (13 core team members). All of the intermediaries also identified their respective government ministry as their primary funding source. On the whole, intermediaries differed more than they were similar with respect to their descriptive characteristics and this lack of commonality contributes to intermediaries continuing to be a “fuzzy” construct.

Interestingly, there was a high level of consistency in the strategies employed by the intermediaries, despite the large variation in intermediary structure and organizational characteristics stated above (Table 5). We did, however, observe a qualitative difference in where the emphasis of the activities was placed across implementation strategies. For example, Te Pou placed a relatively high emphasis on training compared to other activities. The OCoECYMH had a strong emphasis on lived experience and family-targeted activities. The PSSP had the most well-developed link to the synthesis and translation system through EENet and the number of researchers on staff. School Mental Health ASSIST had a strong emphasis on leadership development and capacity-building for mental health within schools and at the school board level. Finally, Mission Mental Health placed a great deal of emphasis on consultation and technical assistance, although not directed toward a particular EIPP, instead, responding to needs identified by the local authorities and regions. Te Pou also had the most well-developed information management strategy, by having national responsibility for managing two data collection systems on behalf of the Ministry of Health. They were followed closely by PSSP, that hosts an information management system for the addictions sector and has been expanding its functionality to support other EIPPs.

**Table 5:** Implementation strategies used by intermediaries by target and by case.

Target	Implementation strategy	Powell et al. (2012) Typology	Use of strategy by case				
			New Zealand	Ontario			Sweden
			Te Pou & Matua Raki	Ontario Centre of Excellence for Child and Youth Mental Health	Provincial System Support Program	School Mental Health Assist	Mission Mental Health
Synthesis and translation system	Developing and disseminating products and tools to support the use evidence in policy/practice	Educate strategy - develop materials (develop effective educational materials) - educate (distribute materials)	✓	✓	✓	✓	✓
	Conducting research and/or contracting with researchers/research organizations	Plan strategy - develop relationships (develop academic partnerships) Quality management strategy - use data experts - capture and share local knowledge	✓	✓	✓	✓	✓
	Bringing exemplars of best practice/evidence from other provinces or countries	Educate strategy - develop materials - educate - educate through peers	✓	✓	✓	✓	✓
	Supporting capacity development for knowledge exchange/implementation	Plan strategy - build buy-in (identify and prepare champions; involve patients/consumers and family members) Educate strategy - develop materials (related to knowledge exchange/implementation)	✓	✓	✓	✓	✓
Delivery system	Training	Educate strategy - educate (develop educational meetings; conduct ongoing training; make training dynamic)	✓	✓	✓	✓	✓
	Consultation and technical assistance	Educate strategy - educate (provide ongoing consultation) Quality management strategy - centralize technical assistance	✓ limited	✓	✓	✓	✓
	Quality assurance/quality improvement	Quality management strategy - develop and organize quality monitoring systems - develop tools for quality monitoring	✓	✓	✓	x	✓
	Leadership development/capacity-building	Plan strategy - initiate leadership (recruit, designate or train for leadership)	✓	✓ limited	x	✓	✓ Goal, but no direct program
	Audit and provide feedback	Quality management strategy - audit and provide feedback	x	x	x	x	x
Other support system	Developing partnerships (with other intermediaries or support system infrastructure)	Plan strategy - develop relationships (build coalitions)	✓	✓	✓	✓	✓
	Undertaking collective action amongst support system infrastructure related to implementation	N/A	✓	✓	✓	✓	✓
Policy system	Formal advice/policy input	N/A	✓	✓	✓	✓	✓
	Informal linkage & exchange with policy makers	N/A	✓	✓	✓	✓	✓
	Bringing forward new policy ideas/system improvements	N/A	✓	✓	✓	✓	✓
	Providing feedback to government on implementation activities/barriers/challenges	N/A	✓	✓	✓	✓	✓
Public	Public awareness/ education	Educate strategy - inform and influence stakeholders (use mass media)	x	x	x	x	x
Lived experience & family	Engaging PWLE and families in activities of intermediary	Plan strategy - build buy-in	✓	✓	✓	x	✓via partner
	Developing tools/resources/training for PWLE and families	Educate strategy - develop materials (develop effective educational materials) - inform and influence stakeholders (prepare patients/consumers to be active participants)	✓	✓	✓	✓	x
Performance assessment/system-monitoring	Hosts data collection system(s)	Quality management strategy - develop and organize quality monitoring systems - use data warehousing techniques - use data experts - capture and share local knowledge	✓	x	✓	✓	x

Despite these differences in emphasis, it is remarkable that there is so much similarity in terms of the implementation strategies employed by the intermediaries given the variation in the mandates and other structural and organizational features. It is also notable that none of the intermediaries used strategies that directly targeted the public (i.e., public awareness and education) or used audit and feedback as a Delivery system strategy. This emerged as the principal policy puzzle that needed to be explained. Specifically, why do these policy intermediaries consistently choose not to engage in these two implementation strategies?

### What explains the lack of use of particular implementation strategies?

Our analysis indicates that there are five reasons why the implementation strategies targeting the public and audit and feedback are not employed by the policy intermediaries: (1) their need to build and maintain healthy relationships with policy actors (public strategies); (2) their need to build and maintain healthy relationships with service delivery system actors (audit & feedback strategy); (3) role differentiation with other system actors (public strategies); (4) lack of “fit” with the role of policy intermediaries (public and audit & feedback strategies); and (5) resource limitations that preclude intensive distributed (program-level) work (audit & feedback strategy).

The first three of these reasons are aspects of Interests using the 3I + E framework. In particular, the role of intermediaries necessarily means they must develop and manage effective relationships with other system actors and as such, they must be highly sensitized to actions that may have a compromising effect on these relationships. The power held by other system actors, and in particular, policy actors in government and service delivery system actors, is exerted indirectly on the intermediaries, what Lukes (52) calls the second dimension of power, causing them to anticipate what strategies would or would not be considered acceptable to those in power and to avoid strategies that could be damaging to these relationships.

For government and policy actors, publicly targeted strategies can sometimes be viewed as supporting advocacy, and advocacy in turn can be perceived by government actors as directly pressuring the government to make changes. Because policy intermediaries often depend on government in multiple ways (e.g., as a funding source, as an implementation partner, as a target of their activities, etc), they prefer to remain as neutral as possible, being perceived as an “honest broker” or a vehicle that enables implementation, rather than specifying what should be implemented. Thus, while the policy actors have not specifically limited the implementation activities of the intermediaries, these intermediaries have shaped their activities to avoid those public-facing strategies that could compromise their relationships with policy actors.

The “honest broker” framing extends to the relationships intermediaries must cultivate with service delivery actors. In order to facilitate implementation, intermediaries must become a trusted source of implementation support for organizations, programs and individual professionals who deliver mental health services to citizens. To build this trust, they prefer implementation strategies that are perceived as facilitative rather than those that may be perceived as more of a performance monitoring or a “watchdog” function. Audit and feedback, when used at the clinical level or at a systems level (e.g., public reporting) can be perceived as falling into the performance-monitoring category and thus, is not a preferred strategy of these intermediaries. Interestingly, some of these intermediaries still play a role in other performance monitoring strategies, by collecting data on behalf of the service delivery system. However, even when they are responsible for this strategy, their approach is often focused on enabling the service delivery sector to use its own data for improvement, or to provide policy makers with additional context for appropriate interpretation of the data and tend not to engage directly in public reporting.

The lack of “fit” of both public strategies and audit and feedback, falls under the Ideas element of the 3I + E framework. This relates to the normative assumptions held by intermediaries and their stakeholders about what policy-focused intermediaries “should” be doing and where there are seen as adding value (and conversely, where they aren't). Finally, past policies (including deinstitutionalization and decisions to offer mental health services across a continuously expanding range of service environments) makes the institutional landscape of mental health services in all three cases large in number and complex for implementation efforts at scale. All of the intermediaries face capacity constraints related to time and money. The strategy of audit and feedback can be cost and time intensive when applied at the individual program level and the intermediaries in our study did not feel they could accomplish this strategy effectively with their existing resources and scope of activity.

## Discussion

Our study sheds further light on policy intermediaries supporting the implementation of EIPPs across mental health systems. These findings help to advance our understanding of the factors that lead to the development of intermediaries in terms of the problems (e.g., negative events involving people with mental illness), policies (e.g., feedback on effectiveness of existing policies) and political events (e.g., changes in government) that are salient in each case. It also presents an in-depth description of the similarities and differences in intermediary structure, organization and use of implementation strategies (e.g., the wide range of structures and organizational mandates contrasting with the striking similarities in terms of implementation strategies employed). Finally, our study provides five reasons why these intermediaries do not use audit and feedback or strategies targeting the public in their work, drawing from explanatory frameworks.

Beyond the contribution of further understanding of intermediaries and their role in facilitating implementation, this study contributes to the literature in two ways. First, our study answers the call made by Nilsen (53) and others to integrate the field of policy implementation with the field of implementation science. We did this by drawing on established theories from political science and through our focus on policy intermediaries. While we found that using these theories was not always a perfect “fit” with questions that relate to the implementation phase of the policy cycle, they were useful in generating unique insights that would not be available from implementation science. Second, we have noted that the vast majority of the literature on intermediaries, and those focusing on mental health and addictions in particular, come from the USA, which has health and social system arrangements that are fairly unique in the world. Our study expands the focus to policy intermediaries in three other countries that each have their own unique health and social system arrangements.

The pre-existing relationship that one author (HB) had with the intermediaries and other system leaders was both a source of strength in this study and a potential limitation. First, these relationships allowed for an IKT approach to the research and likely contributed to the strong response and participation in all three cases. However, her familiarity with the individuals, and her previous role in Ontario and internationally may have influenced how stakeholders responded in the interviews. For example, there were several instances when participants referenced previous conversations or knowledge that HB had and she was sometimes referenced as an influential actor in the development of the intermediaries. Conversely, this familiarity and being established as credible and knowledgeable, may have also meant that participants were more honest, or were likely to delve into issues with greater detail than with an unknown interviewer.

We faced two key challenges with our research. The first relates to the fact that there were no fluent Swedish speakers on the research team. We expect this could have affected the choice of words and phrases participants used in the interviews as well as limiting our ability to use triangulation of sources because many documents were not available in English. The second relates to conducting research in three constantly evolving systems. Since the data collection period, the research team has already noted some shifts in the intermediaries and their contexts making it difficult to be both precise and “current” in our analysis. The ability to adapt and change is likely an important trait for intermediaries and can offset the inherent instability that has been identified as problematic in existing literature (29) but presents a moving target for researchers.

Our study focused on a small number of intermediaries that best fit our definition, yet it was abundantly clear that the infrastructure needed for implementation efforts at a systems level is much more comprehensive. Many more organizations and programs were engaged in mental health policy implementation efforts in these jurisdictions. Some examples include the health quality bodies in New Zealand and Canada and the public health agency in Sweden. Future studies could examine the full complement of infrastructure and how different systems differentiate the implementation strategies among actors. An additional distinction that merits future exploration is the main funding sources and placement of intermediaries across settings (such as government, public sector and private sector), specifically, whether and how the proximity to legislative and regulatory restrictions affects intermediary functions.

Future studies could also use these findings as a foundation from which to build a quantitative study examining a larger number of intermediaries divided among the three sub-types (KT, practice and policy intermediaries) and explore whether and how the use of implementation strategies varies according to sub-type or which strategies are most closely tied to intended outcomes. For example, do policy intermediaries collectively rely on a different subset of implementation strategies than those focussed on implementation in practice settings? Furthermore, the role division and functions of individual team members within an intermediary organization requires further study. Working in a team environment may offset some of the challenges individuals face such as role conflict and ambiguity (29), but role distinction and specialization likely becomes more important (23). How can these roles be optimized in intermediary team settings?

## Conclusions

Policy makers and other actors seeking to implement EIPPs must consider the capacity needed to do it effectively. Our study identifies how intermediaries can be developed and harnessed to support implementation and offers a number of transferrable lessons to those in other jurisdictions. When looking to build implementation infrastructure, policy makers and implementers should make explicit choices in terms of design, with appropriate consideration of the political system context and the health and social system context. They must also pay careful attention to the role of other actors in the system to ensure the intermediary(ies) add value and are optimized to work with those actors effectively. Finally, they should make active decisions about the implementation strategies they intend to employ and monitor their use and effectiveness. To date, much of the focus in implementation science has been at the intervention level, or on the implementation strategies and organizational contexts in which implementation occurs. We forward that it is equally important to consider the vehicles through which these strategies are delivered at scale in systems. This examination of policy intermediaries in mental health systems contributes to this gap in knowledge and increases our understanding of the role intermediaries play in implementation.

## Data Availability

The datasets presented in this article are not readily available because the data were derived from qualitative interviews. Individual transcripts may be identifiable when viewed in whole. Requests to access the datasets should be directed to bullochl@mcmaster.ca.

## APPENDIX

### Interview guide for case study interviews

#### Ethical considerations

A description of the study will have been presented during the recruitment phase. A signed confirmation of commitment to participate will be obtained prior to engaging in the questions. Any ethical issues arising will be addressed prior to the first question and will be documented by the Interviewer.

#### Process

Interviews will be recorded on a digital audio device or computer, transcribed, and uploaded into a qualitative software program. Hand written notes will also be made by the interviewer into her field notebook.

✓ Denotes probes

Date:

Time:

Place:

Interviewer:

Interviewee:

Position of Interviewee:

#### Questions

Do you have any questions for me before proceeding to the interview?

Before we start, I wanted to mention that we will be using the term “mental health” to refer to fields of “mental illness”, “addictions”, “behavioural health” and “health promotion and prevention of mental illnesses and/or addictions” inclusively. It also refers to the health of individuals across the lifespan, not just at particular life stages. Feel free to point out particular or unique features of any of these depending on how your system is arranged, if you feel they are relevant.

### A—current mental health policy priorities

• Can you tell me a little bit about the current policy priorities in [your jurisdiction] that are being implemented? (top 2–4)
  ✓ When were they identified as priorities?
  ✓ How is the implementation of these priorities governed?
  ✓ How are these priorities financed & funded (if at all)?
  ✓ How are they delivered?
    ▪ Consumer-targeted
    ▪ Provider-targeted
    ▪ Organization-targeted

✓ What system challenges are the policy priorities trying to address?
✓ What outcomes are they meant to achieve?
✓ What organizations/programs/people are responsible for implementing them?

### B—structures supporting implementation of mental health priorities (support system & synthesis & translation system)

• I understand from the previous phase of my study that [organization or program] has a role in supporting the implementation of some of the mental health strategic directions/policies/ targets. Can you tell me a little more about them?
  ✓ Who gave them this responsibility?
  ✓ Can you describe [organization or program]’s role and how it functions?
  ✓ What is its size? (in terms of people and funding)
• Who do they provide these activities to? (recipients)
• Do organizations/programs/people from communities voluntarily come to [organization/program] to access implementation supports or does [organization/program] proactively approach the organizations/programs/people in the community? (push vs. pull)
• How are they perceived by other organizations/programs/people in your system?
• Are there other organizations or programs that also play a role supporting the implementation of mental health priorities?
  ✓ Do they differ from [organization or program]? How?
    ○ Generating guidelines
    ○ Generating research & synthesizing it (not just primary research)
    ○ Data systems
    ○ Continuing education

### C—delivery methods and approaches to change being utilized

• What types of activities does [organization/program] engage in? (list from EBSIS, HSE, Franks & Bory & phase 1 of this study)
  ✓ General capacity building
  ✓ Knowledge translation/exchange/mobilization
  ✓ Specific implementation supports (e.g., for a particular Evidence Based Practice, also called technical assistance)
  ✓ Education and training
  ✓ Coaching
  ✓ Research & Evaluation
  ✓ Quality improvement
  ✓ Convening people (in-person/virtual)
  ✓ Consultation
  ✓ Policy & Systems Building
  ✓ Best practice model development
  ✓ Public awareness and education
  ✓ Opinion leaders
  ✓ Audit and feedback
  ✓ Train-the-trainer
  ✓ Other
• Are the activities targeted at the organizational level, the provider level or the consumer/patient level?
• What is the frequency with which they provide these activities?
• Are the people who deliver these activities from [organization/program] located in the communities in which they are delivered? If not, where are they from? (central vs. regional)
• Are there any particular over-arching methods or approaches the [organization or program] utilizes?
  ✓ Implementation science (IS) and the specific IS model
  ✓ Getting-To-Outcomes
  ✓ Quality improvement methods such as LEAN or the IHI model
  ✓ Any other specific methods or approaches that you haven’t already mentioned?

### D—value, challenges & outcomes

• Do you have a sense of what the strengths of this structure and methods might be?
  ✓ Institutions (e.g., government structures, policy legacies, networks), interests (e.g., citizen groups, professional associations, etc), ideas (e.g., values, research, etc)
• Do you feel [organization or program] is valued by the system?
  ○ Who in the system values them?
  ○ Why?
• What are some of the barriers or challenges that are faced in this work?
  ✓ Institutions (e.g., government structures, policy legacies, networks), interests (e.g., citizen groups, professional associations, etc), ideas (e.g., values, research, etc)
• Is [organization/program] able to help achieve the identified policy goals?
• Are there evaluation or outcome data available?

Prompt for documents, presentations or other items that might address any of the topics discussed
